# Biomarkers predicting the effectiveness of normobaric altitude training

**DOI:** 10.1007/s00421-025-06088-3

**Published:** 2025-12-06

**Authors:** Simon Klügel, Svenja Nolte, Sebastian Hacker, Tom Kastner, Nico Walter, Daniel Fleckenstein, Celina Maier, Kristina Gebhardt, Angela Relogio, Deeksha Malhan, Anna Klemmer, Tobias Stauber, Karsten Hollander, Karsten Krüger

**Affiliations:** 1 Department of Exercise Physiology and Sports Therapy, Institute of Sport Science, Justus-Liebig-University Giessen, Kugelberg 62, 35394 Giessen, Germany; 2 Institute of Interdisciplinary Exercise Science and Sports Medicine, Medical School Hamburg, Hamburg, Germany; 3 Institute for Systems Medicine, Medical School Hamburg, Hamburg, Germany; 4 Institute for Molecular Medicine, Medical School Hamburg, Hamburg, Germany; 5 Department of Sports Medicine, Charité - Universitätsmedizin Berlin, corporate member of Freie Universität Berlin and Humboldt-Universität zu Berlin, Berlin, Germany; 6 Institute for Applied Training Science, Leipzig, Germany

**Keywords:** Hypoxia, Athletes, Monocytes, NLR, Iron metabolism, Endurance performance

## Abstract

**Purpose:**

The aim of the present study was to identify blood-based biomarkers that could predict individual VO_2_max improvement before the start of an artificial altitude training camp.

**Methods:**

In an exploratory intervention study, 15 young highly trained athletes from the German Athletics Federation completed a 21-day Live High – Train Low program at an artificial altitude house, which simulated an altitude of 800 km·h (approximately 1.900–2.500 m) under normobaric hypoxia. V̇O₂max was measured pre- and post-intervention, and blood parameters were collected at six time points (pre, post and four times at altitude. The pre measurement functioned as baseline and was used for the predictive model.

**Results:**

Altitude training led to a mean V̇O₂max increase of 2.1 mL · kg⁻¹ · min⁻¹ (+ 3.1%), with high interindividual variability (–1.7 to + 4.5 mL · kg⁻¹ · min⁻¹). Neither training load, sex, nor discipline explained these differences. Logistic regression analyses identified monocyte percentage (> 10%) and neutrophil percentage (> 50%) emerged as the strongest predictors of responder status (McFadden’s R² = 0.61 and 0.65, respectively), while neutrophil-to-lymphocyte-ratio (NLR) and hematocrit also showed significant predictive value. Threshold analyses revealed that high-responders consistently displayed lower neutrophil activity (NLR < 1.5) and higher hematocrit levels (> 42.5%), in contrast to low-responders. Longitudinal biomarker trajectories further supported these distinctions, with high-responders maintaining adaptive immune and iron-related profiles, whereas low-responders exhibited persistent signs of innate immune activation and suboptimal erythropoietic status.

**Conclusion:**

These results highlight the relevance of functional biomarker profiles for individualized training planning in elite sports. Practically, this opens new possibilities for the targeted use of altitude training while minimizing health risks.

**Supplementary Information:**

The online version contains supplementary material available at 10.1007/s00421-025-06088-3.

## Introduction

Altitude training has long been a cornerstone of endurance optimization in elite sports. Among the various approaches, the “Live High - Train Low” (LHTL) paradigm - living in hypoxic conditions while maintaining high training quality under normoxia - has mostly demonstrated performance-enhancing effects through hematological and metabolic adaptations (Chapman et al. 2014; Gore et al. 2013). The physiological rationale behind altitude training is based on the reduction in inspired partial pressure of oxygen, which leads to decreased oxygen availability in tissues. The resulting tissue hypoxia activates a cascade of molecular signaling pathways, triggering both short-term and long-term compensatory mechanisms and thereby inducing adaptive processes in the body. In addition to adaptations within the cardiopulmonary system and acid–base balance, activation of hypoxia-inducible factors (HIFs) stimulates erythropoiesis, resulting in an increased erythrocyte count and total erythrocyte volume. This leads to an elevation of total hemoglobin mass (tHb mass), which is considered a key adaptive mechanism for improving maximal oxygen uptake (V̇O₂max). Typically, these adaptations may be accompanied by improvements in blood buffering capacity, which, alongside V̇O₂max, are considered important predictors of performance in both middle- and long-distance endurance events (Saunders et al. 2019). However, findings on changes in buffering capacity are inconsistent, with some studies reporting improvements (Gore et al. 2001) and others showing no change (Clark et al. 2004; Nordsborg et al. 2012). However, the individual response to exercise training combined with hypoxic stimuli is strikingly heterogenous and a critical issue remains unresolved: which athlete truly benefits from altitude training? While some athletes experience substantial gains in V̇O₂max, others show negligible improvements or even reduced performance (Chapman et al. 1998). Understanding and predicting this variability is paramount, especially considering the substantial logistical, financial, and physiological demands associated with altitude training camps.

In recent years, normobaric hypoxia has emerged as a compelling alternative to traditional hypobaric altitude training. By simulating hypoxic conditions through oxygen-reduced environments at normal atmospheric pressure, normobaric hypoxia allows controlled exposure without the logistical challenges of high-altitude environments (Coppel et al. 2015). Although the physiological stimuli are broadly comparable, underlying key pathways such as HIF-1α-mediated erythropoiesis and metabolic reprogramming, important differences, particularly in oxidative stress and ventilatory responses, have been documented (Faiss et al. 2013).

At the core of adaptation, monitoring and prediction lies the use of biomarkers, measurable biological indicators that reflect physiological processes, adaptive responses, or pathological changes. Biomarkers span a broad range of molecules, including proteins, cells, metabolites, and DNA fragments, and are widely used in medicine for diagnostics, prognostics, risk stratification, and continuous patient monitoring (Ballman 2015; Hacker et al. 2023). Beyond their established clinical applications, biomarkers also play a vital role in sports science as tools for monitoring training load, recovery status, and adaptive capacity (Haller et al. 2023a).

The integration of biomarkers into performance monitoring allows for a shift from reactive to proactive training management, enhancing individualized training prescriptions and reducing injury or maladaptation risks. In the context of altitude training, where physiological strain and adaptation should be finely balanced, biomarker-based monitoring could be pivotal for identifying athletes with the biological prerequisites for successful adaptation (Haller et al. 2023b).

The present exploratory study investigates the predictive potential of specific blood biomarkers, particularly immune and iron-related markers, for individual responsiveness to normobaric altitude training. These biomarkers were selected to represent key mechanisms relevant to altitude adaptation, including erythropoietic regulation (e.g., erythropoietin). (Muckenthaler et al. 2020; Nolte et al. 2025), immune and inflammatory balance (e.g., cytokines, leukocyte profiles, myeloperoxidase) (Khalafi et al. 2023), and factors linking metabolic stress and recovery (e.g. VEGF) (Ramakrishnan et al. 2014). By combining performance diagnostics with detailed biomarker profiling, we aim to deepen the understanding of physiological readiness for adaptation and offer practical insights into precision coaching for elite athletes.

## Materials and methods

This secondary analysis is based on the same dataset and cohort as described by Nolte et al. (2025), aiming to identify predictive blood-based biomarkers. Endurance athletes (running & race walking) from the German Athletics Federation (Deutscher Leichtathletik Verband, DLV) were enrolled in the study. (*n* = 15; females = 9, males = 6; mean age: 19.3 ± 1.4 years; V̇O₂max: 61.40 ± 8.28 mL · kg⁻¹ · min⁻¹). Their disciplines included middle- and long-distance running, marathon, and race walking. Recruitment was carried out in close collaboration with the athletes’ coaches, targeting members of the junior and development squads for the 2023 season. All participants gave written informed consent prior to enrollment. The study received approval from the local ethics committee (MSH-2022/211).

Training was conducted over 21 days at the Höhenhaus Herxheim under normobaric hypoxia. Athletes spent at least 16 h daily in hypoxic conditions (“Live High”) while training under hypoxia or normoxia in the surrounding area (“Train Low”). The cumulative hypoxic dose over the camp was ≥ 800 km·h, expressed as kilometer-hours (equivalent altitude in km × exposure time in hours). The simulated altitude ranged from approximately 1900 to 2500 m, corresponding to an inspired oxygen fraction (FiO₂) between 14.9% and 16.2%. Exact values for FIO₂ and barometric pressure were not recorded, but the facility settings followed standard configurations for moderate altitude exposure used in endurance training. The optimal altitude for such interventions remains a subject of debate; however, altitudes between 2000 and 2200 m above sea level appear to offer a favorable balance between hypoxic stimulus and training quality (Garvican-Lewis et al. 2018; Sharma et al. 2019; Sperlich et al. 2016).

### Exercise testing and training load monitoring

Performance diagnostics were conducted at least one week before and after the training camp to assess V̇O₂max, velocity at V̇O₂max, and maximal running speed to exhaustion (ramp test; 1 min stages; 0.15 m/s increments; 0% incline; MetaLyzer 3B-R2, CORTEX, Leipzig, Germany).

During the three-week altitude training camp, training was planned individually by coaches and athletes, due to the high-performance sports setting. To quantify individual training load, we recorded the session rating of perceived exertion (sRPE) for every training session (Foster et al. 2001; Haddad et al. 2017). Session load was calculated as the product of the athlete-reported RPE immediately post-session and the session duration in minutes (sRPE = RPE × duration). Loads were aggregated per athlete daily, and a mean value was computed across the entire stay in normobaric hypoxia. To contextualize potential changes in load, sRPE-derived training load from the four weeks preceding the altitude camp was also calculated. Analogous to the acute: chronic workload ratio (ACWR), we derived a ratio comparing mean load during the altitude camp with the mean four-week pre-camp load. This metric, termed the Training Load Ratio Altitude (TLRA), served as an index of relative load change (TLRA > 1 indicating an increase; TLRA < 1 indicating a decrease).

## Blood sampling and biomarker analysis

Venous blood was collected at six time points (pre, Day 1, Day 7, Day 14, Day 21, post) between 8 and 10 a.m. following a moderate training session performed on the previous day, approximately 12 h prior to sampling. Hematological parameters (erythrocytes, leukocyte subsets, reticulocytes, hemoglobin, hematocrit, thrombocytes) were analyzed by standard clinical methods (SYNLAB Medical Care Center, Germany). Iron metabolism markers (ferritin, transferrin, transferrin saturation, hepcidin, soluble transferrin receptor, ferritin index (sTfR / log Ferritin)) were likewise assessed. Erythropoietin (EPO) was quantified via ELISA (Thermo Fisher Scientific) with duplicates measured on an Infinite M Plex plate reader (TECAN).

**Fig. 1:**
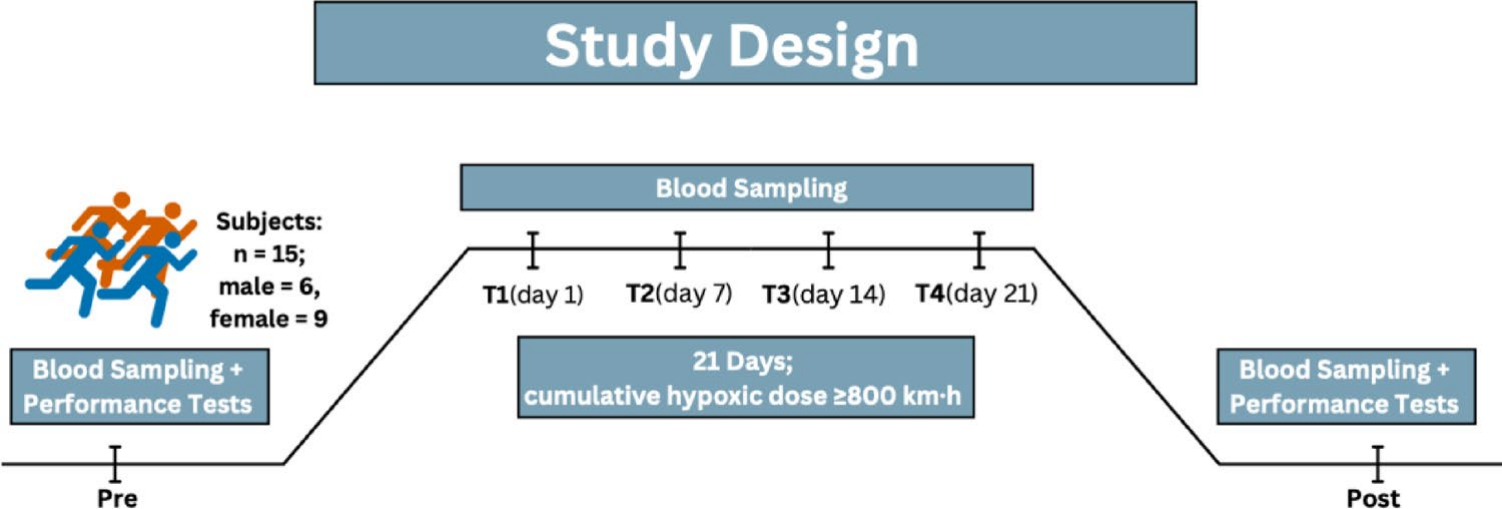
Study design and sampling schedule for the 21-day Live High–Train Low (LHTL) intervention under normobaric hypoxia. Blood sampling occurred at 6 different time points, performance tests were conducted prior and after the intervention. Parameters from the pre measurement were used as baseline and predicting parameters

Immune profiling included absolute leukocyte counts (monocytes, neutrophils, lymphocytes) and their relative proportions, neutrophil–lymphocyte ratio (NLR), lactoferrin, cytokines and growth factors (Interleukin-6 (IL-6), Interleukin-8 (IL-8), Interleukin-10 (IL-10), Tumor necrosis factor-alpha (TNF-α), Vascular endothelial growth factor (VEGF), Interleukin-1 beta (IL-1β), Interleukin-1 receptor antagonist (IL-1ra), Brain-derived neurotrophic factor (BDNF)) and Myeloperoxidase (MPO), measured using magnetic Luminex assay (Bio-Techne Ltd) on the LX-200 platform (Fig. 1).

### Statistical analysis

Data collection was completed using Microsoft Excel (Version 16.41), and statistical analyses were performed using R (Version 4.4.2).

According to the methodology of Chapman et al. (1998), the cohort was retrospectively divided into low- and high-responders based on individual changes in maximal oxygen uptake (V̇O₂max delta) relative to the cohort mean. Low-responders were defined as athletes with an increase of less than 2.1 mL · kg⁻¹ · min⁻¹ (*n* = 6; 2 male, 4 female), while high-responders exhibited increases greater than 2.1 mL · kg⁻¹ · min⁻¹ (*n* = 6; 4 male, 2 female). This cut-off was selected as an indicator of above-average performance improvements attributable to altitude training.

Minor missing data points, resulting from laboratory analysis errors, were imputed using a robust linear regression method, to complete descriptive time-course summaries only. To assess changes in V̇O₂max from pre- to post-training, a paired t-test was performed. Group comparisons for baseline characteristics were performed using Wilcoxon rank-sum tests. No correction for multiple testing was applied, as these analyses serve purely descriptive purposes to characterize the sample rather than to test formal hypotheses. For the identification of predictive biomarkers, each parameter measured before the intervention (time point pre) was individually modeled as an independent variable to evaluate its effect on V̇O₂max response. By design, this approach ruled out multicollinearity, as no interactions between independent variables were introduced. Logistic regression was used to assess the predictive potential of each biomarker from the pre measurement (baseline), using the responder classification as the dependent variable. Despite the presence of outliers, logistic modeling was deemed appropriate given the exploratory nature of the study. Similar exploratory approaches have been used previously to investigate relationships between blood biomarkers and physiological adaptations to exercise (Perroni et al. 2020).

## Results

**Fig. 2:**
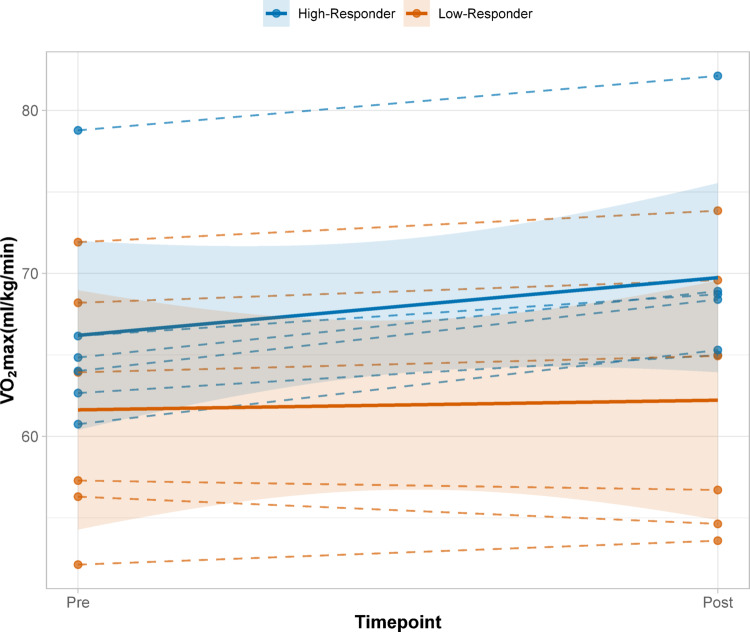
Individual trajectories of V̇O₂max changes and mean trajectories with its standard deviation differentiated by responder status

For subsequent analysis, athletes were retrospectively classified as high- or low-responders based on their individual change in V̇O₂max from pre- to post-intervention. The cohort mean increase (+ 2.1 mL·kg⁻¹·min⁻¹) served as cut-off: athletes with improvements > 2.1 mL·kg⁻¹·min⁻¹ were categorized as high-responders (*n* = 6), while those with < 2.1 mL·kg⁻¹·min⁻¹ were classified as low-responders (*n* = 6). Three athletes were unable to complete post-testing due to injury or illness and were excluded from responder and baseline analyses. Individual responses were highly variable, ranging from a decrease of -1.7 mL · kg⁻¹ · min⁻¹ to an increase of + 4.6 mL · kg⁻¹ · min⁻¹. An average increase of 2.07 mL · kg⁻¹ · min⁻¹ was observed for the total cohort, with a mean increase of 3.54 mL · kg⁻¹ · min⁻¹ in the high-responder group and 0.59 mL · kg⁻¹ · min⁻¹ in the low-responder group (Fig. 2).

Body weight remained stable over the intervention period (pre: 59.43 ± 8.22 kg; post: 59.1 ± 8.1 kg; *p* > 0.05), indicating that performance changes were not confounded by alterations in body mass. Both groups were characterized in anthropometric, performance, hematological and immunological variables. Except monocyte percentage, neutrophil count, neutrophil percentage and lymphocyte percentage no baseline differences were detected (Table 1).

**Table 1:** Baseline characteristics of Low- and High-Responder groups in the primary analysis

Parameter	Low-responder	High-responder	*p*-value
Age (years)	19.00 ± 0.89	19.83 ± 1.72	0.458
Weight (kg)	58.98 ± 8.31	59.88 ± 8.89	0.873
V̇O₂max (ml·kg⁻¹·min⁻¹)	61.63 ± 7.64	66.20 ± 6.44	0.378
V̇O₂abs (ml/min)	3696.00 ± 973.37	3954.83 ± 609.82	0.471
vV̇O₂max (m/s)	5.00 ± 1.13	5.50 ± 0.50	0.517
Leukocytes (10^9/l)	5.39 ± 0.89	4.72 ± 0.91	0.230
Monocytes (10^9/l)	0.41 ± 0.10	0.51 ± 0.10	0.230
Monocytes (%)	7.67 ± 1.59	10.72 ± 0.98	< 0.05 *
Neutrophils (10^9/l)	3.14 ± 0.62	2.10 ± 0.49	< 0.05 *
Neutrophils (%)	58.28 ± 6.77	44.90 ± 7.49	< 0.05 *
Lymphocytes (10^9/l)	1.64 ± 0.39	1.94 ± 0.63	0.575
Lymphocytes (%)	30.47 ± 5.63	40.80 ± 7.84	< 0.05 *
Erythrocytes (10^12/l)	4.51 ± 0.43	4.85 ± 0.29	0.128
Hemoglobin (g/l)	139.50 ± 13.97	146.50 ± 5.79	0.332
Hematocrit (%)	41.45 ± 3.25	45.12 ± 1.15	0.065
Platelets (10^9/l)	237.50 ± 31.79	282.00 ± 51.52	0.199
Reticulocytes (%)	1.23 ± 0.42	0.79 ± 0.23	0.092
Iron (µmol/l)	23.58 ± 11.83	21.98 ± 12.25	0.936
Ferritin (ng/ml)	29.33 ± 16.65	54.62 ± 28.89	0.128
Transferrin (g/l)	2.70 ± 0.34	2.73 ± 0.48	0.936
Transferrin Saturation (%)	42.18 ± 5.75	32.61 ± 9.54	0.093

Data are presented as mean ± standard deviation. Significant group differences (*p* < 0.05) are marked with an asterisk

## Predictive biomarkers and their associations

**Fig. 3:**
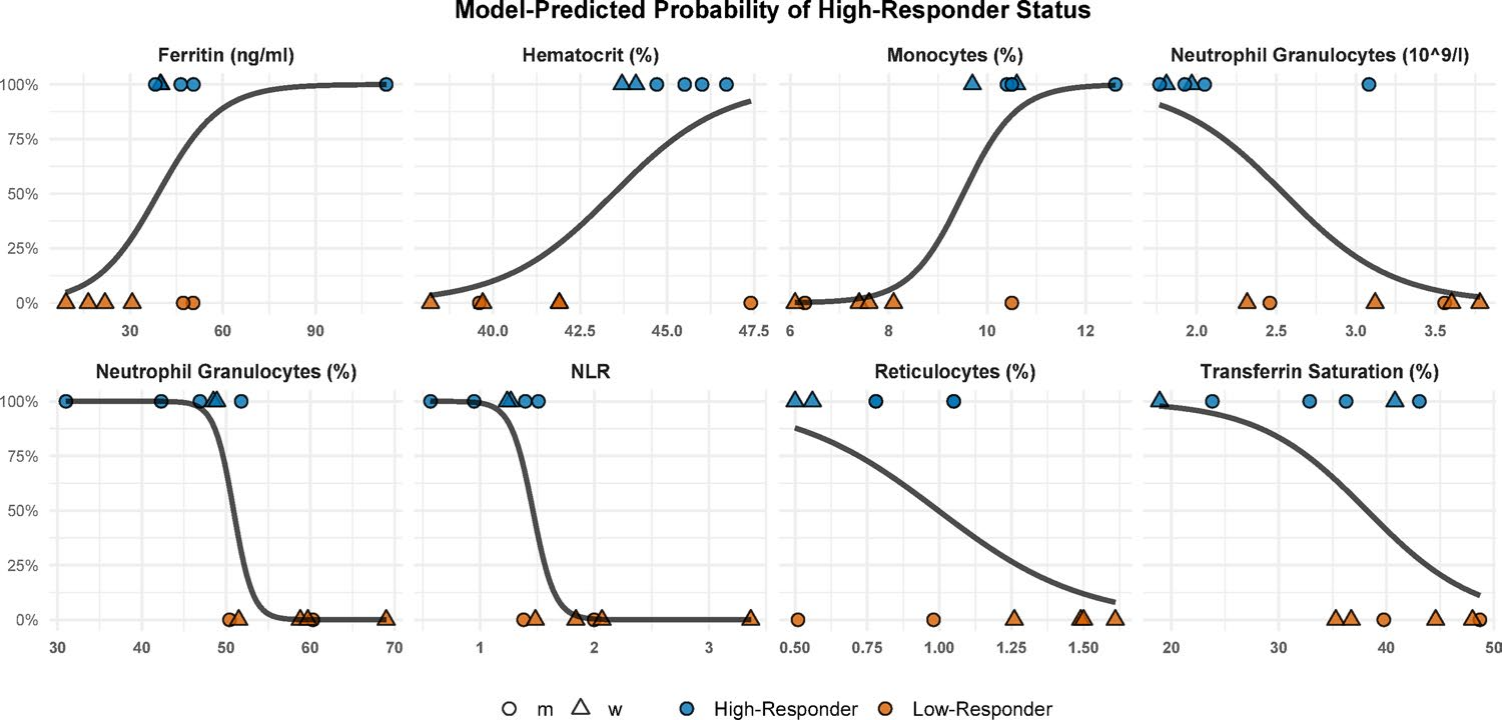
Estimate Plots of Logistic Regression Analyses for Ferritin (**A**), Hematocrit (**B**), Monocytes (**C**), Absolute Neutrophil Count (**D**), Neutrophil Percentage (**E**), the Neutrophil-Lymphocyte-Ratio (**F**), Reticulocytes (**G**) and Transferrin Saturation (**H**). The black curve represents the logistic function curve showing the relationship between biomarker concentration and the probability of above-averaged V̇O₂max improvement (high-responder status)

Logistic regression analyses revealed that neither average training load, discipline, nor sex accounted for responder status, underscoring the need to explore intrinsic biological determinants of adaptation. Robust associations between the biomarker profiles and responder status were found. Monocyte percentage (χ² = 10.16, *p* = 0.0014, McFadden’s R² = 0.61) and neutrophil percentage (χ² = 10.89, *p* = 0.0010, R² = 0.65) emerged as a strong predictor of adaptation. The absolute neutrophil count (10⁹/l) (χ² = 7.49, *p* = 0.0062, R² = 0.42) and the NLR (χ² = 9.80, *p* = 0.0017, R² = 0.59) also displayed significant predictive power (Fig. 3). In the domain of iron-related markers, hematocrit (χ² = 5.70, *p* = 0.0169, R² = 0.34), reticulocyte percentage (χ² = 4.54, *p* = 0.0331, R² = 0.34), ferritin (χ² = 4.68, *p* = 0.0306, R² = 0.28), and transferrin saturation (χ² = 4.46, *p* = 0.0350, R² = 0.27) were identified as significant contributors to predicting training response (Fig. 3). No associations were found for erythrocytes, leukocyte count, lymphocytes, hemoglobin, thrombocytes, transferrin, hepcidin, soluble transferrin receptor, ferritin index, EPO, MPO, IL-6, IL-8, IL-10, TNF-α, VEGF, IL-1ra and BDNF (Supplement).

Threshold analyses demonstrated distinct biomarker patterns that differentiated high- from low-responders. Monocyte percentages above 10% were predominantly found among high-responders, while neutrophil percentages exceeding 50% were exclusively observed in low-responders. Similarly, NLR values below 1.5 and hematocrit levels above 42.5% aligned consistently with above-averaged V̇O₂max adaptations (Fig. 3).

Biomarker profiles of the excluded subgroup showed monocyte percentages of 8.6 ± 2.5%, neutrophil percentages of 56.9 ± 8.5%, NLR of 1.81 ± 0.61, hematocrit of 39.8 ± 2.5%, ferritin levels of 36.7 ± 20.7 ng/mL, and transferrin saturation of 43.3 ± 8.8%.

### Temporal dynamics of biomarker trajectories

**Fig. 4:**
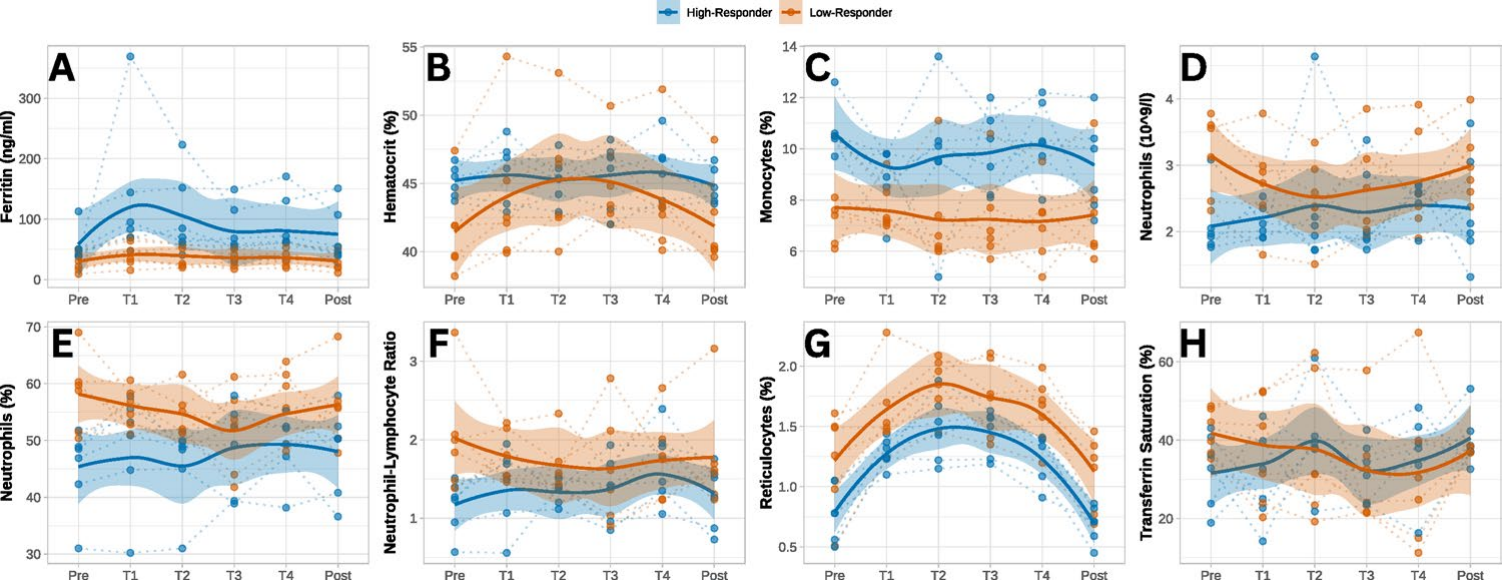
Time Courses of potential predictors: Ferritin (**A**), Hematocrit (**B**), Monocytes (**C**), Absolute Neutrophil Count (**D**), Neutrophil Percentage (**E**), the Neutrophil-Lymphocyte-Ratio (**F**), Reticulocytes (**G**) and Transferrin Saturation (**H**)

Figure 4 illustrates the temporal trajectories of selected hematological and inflammatory biomarkers across six time points (Pre, T1–T4, Post), stratified by responder status. Baseline differences between high- and low-responders were evident across key biomarkers, particularly monocyte percentage, neutrophil percentage, NLR, and hematocrit, with clear group separations that persisted throughout the training period.

High-responders exhibited more stable or adaptive biomarker patterns, with relatively low and stable levels of neutrophils and NLR, and consistently elevated hematocrit and ferritin levels. In contrast, low-responders showed sustained elevations in innate immune markers, especially neutrophils and NLR, throughout the intervention, alongside comparatively lower hematocrit and ferritin levels (Fig. 4).

## Discussion

This exploratory study investigated the potential of blood-based biomarkers to predict individual adaptation to a normobaric altitude training camp in highly trained endurance athletes. Our main finding was that baseline immune and iron-related markers, particularly monocyte percentage, NLR, ferritin, and hematocrit, were significantly associated with V̇O₂max improvements following the intervention. Athletes with elevated monocyte percentage (> 9.5%), low NLR (< 1.6), and favorable iron status prior to altitude exposure demonstrated superior adaptive responses, while others exhibited minimal or even negative changes in aerobic capacity. These results highlight the predictive utility of functional biomarker profiling for identifying athletes with high adaptation potential as well as those at risk for maladaptive responses or health complications, reinforcing the concept that individual biological readiness plays a critical role in the effectiveness of altitude training.

Comparing baseline characteristics between low- and high-responders, all variables except for the lymphocyte percentage differ significantly, mirroring the findings of the regression analysis. Notably, the baseline biomarker profiles of the excluded subgroup (due to illness or injury) support these findings: monocyte counts below the 9.5% threshold, NLR values above 1.6 and suboptimal iron status indicators including low ferritin. Additionally, their neutrophil profiles and hematocrit values aligned with patterns observed in low-responders from the primary analysis. Importantly, temporal analyses revealed that baseline biomarker differences between high- and low-responders were not only stable but also shaped divergent inflammatory trajectories during the intervention, underscoring the interplay between immune status and endurance adaptation.

Monocytes play a key role in the initial response to physical stress, particularly under hypoxic-mechanical conditions (Colgan et al. 2020). They differentiate into various macrophage subtypes that contribute to the resolution of inflammation, tissue repair, and metabolic reprogramming (Timmerman et al. 2008). In skeletal muscle, macrophages fulfill an essential function in angiogenesis and in the recruitment of satellite cell–mediated regenerative processes (Juhas et al. 2018). Moreover, monocytes upregulate the expression of HIF-1α (hypoxia-inducible factor 1 alpha) under hypoxic conditions, a key regulator of the cellular response to oxygen deprivation (Asano et al. 1998). HIF-1α activation induces extensive metabolic adaptations, including increased glycolysis, modulation of mitochondrial activity, and elevated production of growth factors such as VEGF (vascular endothelial growth factor). A higher proportion of circulating monocytes may thus reflect an adaptively responsive immune profile that facilitates the effective integration of hypoxic stimuli. Nevertheless, this interpretation remains speculative, as monocytes constitute a heterogeneous cell population comprising classical (CD14⁺⁺CD16⁻), intermediate (CD14⁺⁺CD16⁺), and non-classical (CD14⁺CD16⁺⁺) subsets with distinct immunological functions (Wong et al. 2012; Ziegler-Heitbrock 2014). Classical monocytes are mainly phagocytic and mediate early inflammatory responses, whereas non-classical monocytes are involved in endothelial patrolling and anti-inflammatory regulation (Patel et al. 2017). The relative distribution of these subsets may therefore influence the immune and adaptive response to hypoxic training stimuli. However, flow cytometric analyses were not available in the present study, preventing us from distinguishing between these subsets. Future studies should therefore include phenotypic characterization of monocyte populations to elucidate their specific contributions to exercise-induced immunological adaptations.

Neutrophil granulocytes are primary actors of the innate immune response and typically increase in the context of acute and chronic inflammatory reactions. A persistent elevation in neutrophil count, even within the subclinical range, has been associated with various adverse physiological processes, including enhanced oxidative stress (Nathan 2006) endothelial dysfunction (Kaya et al. 2014), and impaired mitochondrial biogenesis (Faas and De Vos 2020). These factors could substantially compromise the effectiveness of training-induced adaptations, particularly under hypoxic conditions such as those encountered in altitude training. The threshold of 50% observed in this study may therefore be interpreted as a pragmatic cutoff for predicting favorable training adaptation. Previous research has shown that a chronically elevated neutrophil count, even with moderate increases, provides indications of systemic inflammatory processes that can have long-term negative effects on cardiovascular and metabolic health (Núñez et al. 2008).

Closely linked to this, the NLR also emerged as a meaningful predictor in this study. A low NLR indicates a balanced relationship between nonspecific inflammatory response (neutrophils) and adaptive regulation (lymphocytes). In the literature, a low NLR has been associated with improved cardiovascular function and greater stress resilience (Widasari et al. 2023; Zhu et al. 2020). These aspects are particularly relevant in altitude training, where oxidative and mechanical stress increase at the cellular level. Elevated NLR-levels could indicate insufficient readiness to altitude training adaptation and therefore converting the beneficial hypoxic stimulus into detrimental physiological stress.

A balanced inflammatory state may allow hypoxia-induced signaling pathways, including those involving HIF-1α and erythropoietin, to function effectively without interference from competing inflammatory activity. In this perspective, a low NLR appears not only as a marker of recovery but also as a “gatekeeper” for cellular plasticity and metabolic adaptation (Kramer et al. 2024). At the same time, the data indicate that a low NLR, as an expression of immunological balance, may be linked not only to better exercise tolerance but also to more efficient mitochondrial adaptation. These findings complement current considerations regarding the role of the immune system in training adaptation and shed new light on the relevance of systemic markers in the performance process.

The predictive relevance of ferritin and hematocrit levels aligns with current research on the importance of iron metabolism in altitude training (Stellingwerff et al. 2019). Even mild iron deficiency, without manifest anemia, can limit erythropoiesis and thereby hinder performance development. The recommended ferritin cutoff for altitude training, according to recent literature, ranges between 30 and 50 ng/ml, a range that also corresponds to the threshold identified in the present study (Nolte et al. 2024).

In combination with a functional immune profile, an optimal iron status provides the biochemical foundation for red blood cell production and enhanced oxygen transport capacity. Beyond the specific context of artificial altitude exposure, blood biomarkers as used in this study may also hold promise for predicting individual responsiveness to other forms of exercise training. Integrating traditional biochemical markers with multivariate molecular data and applying advanced bioinformatics approaches, such as weighted gene coexpression network analysis (WGCNA), could further enhance the mechanistic understanding of exercise adaptation. Previous work has shown that gene networks related to mitochondrial and posttranslational processes are preserved between skeletal muscle and circulating leukocytes during exercise recovery (Broadbent et al. 2017), supporting the potential of blood-derived molecular signatures to reflect muscular adaptations and to serve as accessible proxies for systemic training responses. This underscores the necessity of conducting comprehensive blood-based diagnostics prior to altitude training interventions - going beyond simple hemoglobin screening (Hacker et al. 2023). These findings underscore the systemic nature of adaptation: immune status, inflammatory balance, and iron availability interact synergistically to shape the training response. This integrated perspective advances beyond traditional performance diagnostics, offering a foundation for personalized training strategies in elite sport.

This exploratory study is limited by its sample size and retrospective responder classification. Validation in larger cohorts and inclusion of additional molecular markers, such as transcription factors, metabolic profiles, and cellular stress indicators, are warranted. Furthermore, the present study did not differentiate monocyte subsets, which may have provided additional mechanistic insight into the observed associations between immune status and training adaptation. Another limitation of the present study is that the exact barometric pressure and FIO₂ inside the normobaric facility were not recorded. Therefore, the simulated altitude and inspired PO₂ values were estimated based on standard altitude-FIO₂ equivalences commonly applied in similar normobaric hypoxia settings. In addition to the V̇O₂max test, the inclusion of a test race under real-world conditions would have strengthened the assessment of performance improvements following altitude training. Ideally, a standardized biomarker panel like this could be applied prospectively to stratify individuals prior to altitude training, serving as part of a diagnostic dashboard to guide individualized altitude training planning.

## Conclusions

The present data could support a paradigm shift in the planning of altitude training interventions. Rather than applying a “blanket approach”, blood-based markers could be used prospectively to identify athletes who are most likely to benefit from such interventions. This has clear practical implications: pre-altitude training screening should include the assessment of monocytes, neutrophils, NLR, ferritin, and hematocrit as a basis for planning and individualization. Targeted preparation can include identifying and then possibly deciding whether someone should engage in altitude training or not (Haller et al. 2023b). Such an approach can enhance the efficiency of altitude training by enabling better allocation of personnel, time, and financial resources within elite sport. Moreover, it may reduce health risks by identifying athletes with unfavorable conditions who might experience detrimental rather than beneficial adaptations to altitude exposure. By combining performance diagnostics with detailed biomarker profiling, we aim to deepen the understanding of physiological readiness for adaptation and offer practical insights into precision coaching for elite athletes.

## Supplementary Information

Below is the link to the electronic supplementary material.

Supplementary Material 1

## Data Availability

All data is available upon request. Please reach out to karsten.krueger@sport.uni-giessen.de.
