# Supplementary material for: Biomarkers predicting the effectiveness of normobaric altitude training
Source: Eur J Appl Physiol. 2025 Dec 6;126(5):2553–62. doi: 10.1007/s00421-025-06088-3 (PMC13236823; doi:10.1007/s00421-025-06088-3)
Supplement: Supplementary file 1 — Supplementary Material 1 [file 421_2025_6088_MOESM1_ESM.docx]

| Parameter | pre | t1 | t2 | t3 | t4 | post |
| --- | --- | --- | --- | --- | --- | --- |
| Leukocytes (10^9/l) | 5.35 ± 1.06 | 4.9 ± 0.98 | 4.76 ± 1.05 | 4.9 ± 0.98 | 4.79 ± 0.81 | 5.54 ± 1.57 |
| Lymphocytes (10^9/l) | 1.85 ± 0.49 | 1.74 ± 0.59 | 1.76 ± 0.59 | 1.85 ± 0.56 | 1.7 ± 0.46 | 1.89 ± 0.61 |
| Lymphocytes (%) | 35.01 ± 7.87 | 35.11 ± 6.87 | 36.78 ± 7.3 | 37.97 ± 8.11 | 34.96 ± 6.21 | 34.69 ± 7.65 |
| Erythrocytes (10^12/l) | 4.63 ± 0.37 | 4.81 ± 0.46 | 4.92 ± 0.37 | 4.93 ± 0.33 | 4.78 ± 0.45 | 4.68 ± 0.35 |
| Hemoglobin (g/L) | 140.8 ± 10.8 | 149.47 ± 15.3 | 153.4 ± 11.27 | 154 ± 12.13 | 154.34 ± 13.4 | 143.73 ± 10.12 |
| Thrombocytes (10^9/L) | 268.6 ± 53.41 | 246.33 ± 42.46 | 275.4 ± 49.38 | 276.54 ± 50.69 | 272.73 ± 51.88 | 246.93 ± 42.1 |
| Transferrin (g/L) | 2.71 ± 0.38 | 2.73 ± 0.46 | 2.8 ± 0.4 | 2.8 ± 0.39 | 2.79 ± 0.44 | 2.78 ± 0.43 |
| Hepcidin (ng/mL) | 7.88 ± 6.79 | 8.82 ± 10.51 | 9.52 ± 7.58 | 6.21 ± 5.59 | 7.2 ± 6.29 | 7.55 ± 8.05 |
| Soluble Transferrin Receptor (mg/L) | 0.88 ± 0.2 | 1.5 ± 0.54 | 1.46 ± 0.34 | 1.52 ± 0.34 | 1.51 ± 0.26 | 0.79 ± 0.19 |
| Ferritin Index | 0.58 ± 0.13 | 0.81 ± 0.29 | 0.89 ± 0.3 | 0.94 ± 0.28 | 0.95 ± 0.21 | 0.5 ± 0.11 |
| Brain-derived Neurotrophic Factor | 3104.87 ± 2438.63 | 2941.34 ± 1889.85 | 2977.36 ± 1990.22 | 2555.28 ± 1532.1 | 2772.74 ± 1994.15 | 3766.55 ± 2932.29 |
| Myeloperoxidase | 66883.05 ± 80028.06 | 27021.49 ± 15796.25 | 29242.45 ± 20453 | 30344.2 ± 17753.57 | 29393.84 ± 18471.29 | 71828.88 ± 84670.61 |
| Interleukin-10 | 1.47 ± 1.46 | 1.59 ± 1.7 | 1.61 ± 1.63 | 1.57 ± 1.63 | 1.55 ± 1.48 | 2.02 ± 2.02 |
| Interleukin-1ra | 4609.29 ± 11537.3 | 4420.88 ± 11089.31 | 4657.1 ± 11432.38 | 4708 ± 11679.33 | 4301.64 ± 10037.18 | 5187.89 ± 12468.72 |
| Interleukin-6 | 3.43 ± 3.21 | 3.85 ± 3.74 | 3.81 ± 3.63 | 3.76 ± 3.69 | 3.61 ± 3.56 | 4.07 ± 4.15 |
| Interleukin-8 | 2.87 ± 1.93 | 3.05 ± 2.07 | 3.69 ± 2.3 | 3.66 ± 2.36 | 3.84 ± 2.26 | 3.32 ± 2.33 |
| Tumor Necrosis Factor-alpha | 7.56 ± 7.78 | 7.94 ± 8.44 | 7.91 ± 7.98 | 8.26 ± 8.04 | 7.98 ± 7.74 | 7.97 ± 8.12 |
| Vascular Endothelial Growth Factor | 45.27 ± 28.88 | 51.2 ± 41.66 | 62.78 ± 57.59 | 57.19 ± 46.16 | 53.57 ± 39.99 | 53.65 ± 32.83 |
| Erythropoietin | 10.22 ± 4.59 | 15.16 ± 7.44 | 10.84 ± 5.39 | 11.68 ± 5.01 | 10.67 ± 4.62 | 10.47 ± 6.83 |
